# Facteurs associes aux décès des nouveau-nés suspects d'infections bactériennes au Centre Hospitalier Universitaire Pédiatrique Charles de Gaulle de Ouagadougou, Burkina Faso

**DOI:** 10.11604/pamj.2016.24.7.6599

**Published:** 2016-05-04

**Authors:** Solange Odile Yugbaré Ouédraogo, Désiré Méda, Lassina Dao, Fla Kouéta, Kam Ludovic, Ramata Ouédraogo Traoré, Diarra Yé

**Affiliations:** 1 A Unité de Formation et de Recherche en Sciences de la Santé (UFR/SDS), Université de Ouagadougou, Burkina Faso; 2 Centre Hospitalier Universitaire Pédiatrique Charles de Gaulle Ouagadougou, Burkina Faso; 3 Centre Hospitalier Universitaire Yalgado Ouédraogo, Ouagadougou, Burkina Faso

**Keywords:** Nouveau-né, infection bactérienne, bactériologie, facteurs associés, décès

## Abstract

**Introduction:**

Il s'agit d’étudier les facteurs associés au décès des nouveau-nés suspects d'infections bactériennes au centre hospitalier universitaire pédiatrique Charles de gaulle de Ouagadougou.

**Méthodes:**

Nous avons mené une étude de cohorte rétrospective du 1er janvier 2009 au 31 décembre 2012 au centre hospitalier universitaire pédiatrique Charles de gaulle de Ouagadougou.

**Résultats:**

La fréquence hospitalière des nouveau-nés suspects d'infection bactérienne sur était de 62,8%. L’âge médian à l'admission était de trois jours et le sex ratio de 1,1. Parmi ces nouveau-nés, 351 (22,8%) ont bénéficié d'au moins un examen bactériologique, et 28 (8%) ont eu la confirmation de l'origine bactérienne de l'infection. Au cours de la période néonatale, 138(9%) nouveau-nés sont décédés avec un taux de létalité précoce et tardive respectivement de 9,6% et 8,3%. Le lieu de résidence, le mode d'admission, le nombre de consultations prénatales, le poids de naissance, la présence de signes de gravité et l'année d'admission étaient les facteurs de risque indépendants associés au décès.

**Conclusion:**

Les facteurs associés au décès devraient être pris en compte dans les interventions de santé pour réduire la mortalité néonatale.

## Introduction

Dans les pays à forte mortalité comme en Afrique subsaharienne, près de la moitié des décès néonatals est attribuable aux infections [1]. Les infections néonatales regroupent, en général, les sepsis/méningites/tétanos (15%), la pneumonie (11%) et les diarrhées (2%). Au Burkina Faso, elles seraient responsables de 37% des décès néonatals [2]. Au Centre Hospitalier Universitaire Pédiatrique Charles de Gaulle (CHUP-CDG) de Ouagadougou, 73,9% nouveau-nés hospitalisés pour infections néonatales avaient un taux de létalité de 16,8% [3]. La connaissance des facteurs associés aux décès des nouveau-nés suspects d'infections bactériennes serait la base pour renforcer et mieux cibler les interventions de santé pour ce groupe à risque.

## Méthodes

Il s'est agi d'une étude rétrospective de type descriptif d'une cohorte du 1er janvier 2009 au 31 décembre 2012 portant sur les paramètres épidémiologiques, cliniques, bactériologiques des nouveau-nés de 0 à 28 jours, hospitalisés au CHUP-CDG de Ouagadougou pour une suspicion d'infection bactérienne. Les variables suivantes, paternelles, maternelles (état civil, nombre de consultations prénatales, type de grossesse, lieu d'accouchement, voie d'accouchement), néonatales ( réanimation, mode d'admission, année et la saison d'admission, âge au début de la maladie, délai de consultation, signes de gravité, bactériologie, statut à la fin du suivi) ont été spécifiquement étudiées. Les données ont été analysées avec SPSS 21 (Statistical Package for Social Sciences, version 21) et le test de chi- carré a été utilisé pour comparer les variables catégorielles avec un seuil de significativité à 5%.

## Résultats

Nous avons répertoriés 1540 nouveau-nés sur 2449 nouveau-nés hospitalisés soit une fréquence de 62,8%. Les nouveau-nés étaient issus de familles à niveau socio-économique moyen dans la moitié des cas (53,1%) et 92,4% d'entre eux résidaient dans la ville Ouagadougou. Le sex ratio était de 1,1 avec un âge médian à l'admission de trois jours et des extrêmes allant de 0 et 28 jours. L’âge médian du début de la maladie était de deux jours avec des extrêmes allant de 0 et 27 jours. Les accouchements s’étaient déroulés dans la majorité des cas ( 98,4%) dans un centre de santé et très souvent par voie basse ( 89,3%). Le poids moyen de naissance était 2842 grammes avec un écart type de 548 grammes. Les nouveau-nés étaient très souvent référés (70,2%) au CHUP-CDG et avaient au moins un signe de gravité à l'admission (56,4%). Le délai médian de consultation était d'un jour, avec des extrêmes allant de 0 et 28 jours. Quatre cent dix-sept (417) examens bactériologiques ont été effectués chez 351 nouveau-nés et 29 (6,9%) prélèvements sont revenus positifs. Les examens les plus réalisés étaient la culture du liquide céphalorachidien suivi de l'uroculture et de la coproculture avec respectivement 57,6%, 19,6% et 18,7%. Le Tableau 1 donne la répartition des différents germes isolés par les examens bactériologiques. Les germes étaient à 75,9% des bacilles à Gram négatif(BGN) contre 24,1% pour les cocci à Gram positif(CGP). *Escherichia. Coli* (E.coli) et *Klebsiella pneumoniae* étaient isolés dans respectivement 34,4% et 17,2% cas. Parmi les 1540 nouveau-nés suspects d'infection bactérienne, 138 (9%) sont décédés avec des taux de létalité précoce et tardive respectivement de 9,6% et 8,3%. Nous avons observées 33 perdus de vue en 2009, 21 en 2010 et 2011 et 20 en 2012. Le Tableau 2 présente l'analyse bivariée entre la variable dépendante « décès » et les variables sociodémographiques. Le lieu de résidence de la mère (p < 0,001), le niveau socio-économique (p < 0,001), et l’âge au début de la maladie (p < 0,01) sont associés significativement au décès L'analyse bivariée entre le décès et les variables anténatales et périnatales retrouve la consultation prénatale (p < 0,001), la réanimation à la naissance (p < 0,001), et le poids de naissance (p < 0,001), comme facteurs significativement associés au décès (Tableau 3). Le Tableau 4 donne les résultats de l'analyse multivariée des variables associées au décès. La résidence, le poids à la naissance, le mode d'admission, l'année d'admission, la présence de signe de gravité et le nombre de consultations prénatales sont les facteurs indépendants associés à la mortalité néonatale.

**Tableau 1:** Répartition des germes isolés par la bactériologie

Germes		Effectifs				Total	Pourcentage (%)
	Année	2009	2010	2011	2012		
E. coli*			3	5	2	10	34,4
Klebsiella pneumoniae*		2	2	1		5	17,2
Proteus sp*		1		1		2	7
Staphylococcus aureus**			1		1	2	7
Staphylococcus saprophyticus**		1			1	2	7
Streptocoque D**				1	1	2	7
Streptococcus pneumoniae**			1			1	3,4
Enterobacter sp*				1		1	3,4
Salmonella sp*				1		1	3,4
Pseudomonas aeruginosa*		1				1	3,4
Enterobacter cloacae*		1				1	3,4
Citrobacter spp*		1				1	3,4
Total***		7	7	10	5	29	100

* BGN: 75,9%

** CGP: 24,1%

*** un des 28 nouveau-nés ayant confirmé l'infection bactérienne a eu deux prélèvements positifs, d'où 29 germes

**Tableau 2:** Analyse bivariée entre le décès et les variables sociodémographiques

Variables	Effectifs	OR	IC 95%)	p
Niveau socio-économique (n = 1418)				
Moyen	762	1	1,31-2,78	<0,001
Bas	656	1,91		
Résidence de la mère (n = 1445)				
Ouagadougou	1311	1	2,51-6,59	<0,001
Autre	104	4,07		
Age à l'admission (n = 1445)	1445	0,98	0.96-1	0,102
Age au début de la maladie(1406)	1406	0,1	0,9	0,01

**Tableau 3:** Analyse bivariée entre le décès et les variables anténatales et périnatales

Variables	Effectifs	OR	IC95%	p
Consultation prénatale (1400)				
≥4	673	1	1,3-1,8	<0,001
<4	727	1,9		
**Voie d'accouchement (n = 1445)**				
Basse	1284	1,5	0,9-2,5	0,110
Haute	161	1		
**Réanimé à la naissance (n = 1445)**				
Oui	326	0,5	0,3-0,7	<0,001
Non	1119	1		
**Poids de naissance (n = 1280)**	1280	0,9	0,8-0,9	<0,001

**Tableau 4:** Analyse multivariée des facteurs associés au décès des nouveau-nés suspects d'infections bactériennes au CHU-CDG de 2009 à 2012

Variables	Ajustés		Analyse de sensibilité	
	OR	IC95%	OR	IC95%
**Résidence**				
Ouagadougou	1		1	
Autres	2,9	1,7-5	2,6	1,6-3,8
**Mode d'admission**				
Direct	1		1	
Référé	1,9	1,1-3,4	1,6	1,1-2,3
**Age à l'admission**				
0-1	1	0,6-1,8	1,3	0,8-1,9
2-3	0,7	0,4-1,3	0,8	0,5-1,3
4-10	1,2	0,7-2,1	1,4	0.9-2,2
≥10	1		1	
**Consultation prénatale**				
≥4	1		1	
<4	1,7	1-2,4	1,4	1-1,9
**Signe de gravité**				
Non	1		1	
Oui	5,9	3,6-9,3	2,9	2,1-3,9
**Poids de naissance**	0,9	0,9-1	0,9	0,9-1
**Année d'admission**				
2009	1		1	
2010	2,2	0,9-6,4	0,77	0,5-1,7
2011	8,3	3,2-21,8	1,62	1-2,6
2012	10	4-25,8	1,8	1,1-2,7

## Discussion

Au CHUP-CDG la prévalence de l'infection bactérienne néonatale de 62,8% est proche de celles de Dao et al. en 2009 (68,5%) [4] et de Ambe et al (62,3%) au Nigéria [5]. Des prévalences plus faibles ont été retrouvées par certains auteurs: Glorion (17,2%) au Sénégal [6], et en occident: Hersent (4,5%), Noguer et al. (1,7%) en France [7, 8]. La prévalence de l'infection bactérienne est très variable à cause de la définition et du niveau de développement des pays. Les mesures défaillantes d'hygiène et d'asepsie sous nos tropiques en milieu hospitalier et dans l'entourage familial sont des facteurs qui expliqueraient cette différence [9]. Sur les examens bactériologiques réalisés, seulement 6,9% sont revenus positifs Ces résultats corroborent ceux d'autres auteurs africains. Djoupomb Njanang à Yaoundé [10] et Cissé et al. à Dakar [11], qui avaient trouvé respectivement des taux de positivité de 9,6% et 10,6% mais restent inférieures à celles de Glorion à Dakar (20,8%) [6], Dao et al. (28,5%) [4]. Ces différences pourraient s'expliquer par les types de prélèvements. Ainsi, dans l’étude de Dao et al., il y avait plus de coprocultures et de cultures de sérosités qui ont un taux de positivité plus élevé [4].

D'une manière générale, la preuve de l'infection bactérienne chez le nouveau-né demeure difficile à établir. Cependant la répétition des examens donneraient plus de chance d'isoler le germe. En réalisant quatre hémocultures et deux coprocultures chez chaque nouveau-né à Madagascar, Andrianarivelo et al ont retrouvé une proportion de positifs de 52,4% [12]. Aussi, des examens comme le prélèvement gastrique contribuerait plus à isoler le germe [13]. Les bacilles à Gram négatif (BGN) représentaient 75,9% contre 24,1% pour les cocci à Gram positif (CGP). La prédominance des BGN a été retrouvée dans d'autres études africaines. Djoupomb Njanang à Yaoundé (85,8%) [10], Cissé et al.à Dakar (85,8%) [11]. Dans les études africaines, les germes les plus retrouvés sont *E. coli, Klebsiella, Staphylococcus* dans des proportions variables [4, 5, 10, 12], alors que dans les pays occidentaux, prédominent le Streptoccoque du groupe B [14] et *Listeria monocytogenès* [15] Les conditions précaires d'asepsie et la proportion élevée des infections bactériennes précoces expliqueraient la prédominance de ces germes dans les études africaines. En effet *Klebsiella* et *Staphylococcus*, bien que présent dans les infections maternofœtales (IMF), sont des germes opportunistes retrouvés dans l'environnement et à la surface des muqueuses [9, 13, 16] tandis que E. coli, classiquement en cause dans les IMF, est de plus en plus responsable d'infections nosocomiales par une hygiène défectueuse [17, 18]. La létalité des nouveau-nés suspects d'infection bactérienne dans notre étude était de 9%. Notre taux est inférieur au taux global de mortalité hospitalière néonatale au CHUP-CDG, au Burkina et dans des études hospitalières en Afrique [5, 10, 19]. Cette létalité moindre serait attribuable à la mise en œuvre de la stratégie de réduction des soins obstétricaux et néonatals d'urgence par le gouvernement depuis 2006 [20]. Le poids à la naissance, le lieu de résidence, l'année d'admission, le nombre de consultations prénatales, le mode d'admission et la présence de signes de gravité étaient les facteurs indépendants associés significativement au décès chez les nouveau-nés suspects d'infections bactériennes. Notre étude montre que la quasi-totalité des facteurs associés à la mortalité des nouveau-nés suspects d'infections bactériennes se retrouvent également dans d'autres études menées dans les pays en développement et surtout africaines [21–24].

Beck, au Rwanda, dans une étude sur l’évolution des facteurs associés aux tendances de mortalité néonatale, a retrouvé que les nouveau-nés, dont les mères avaient faits au moins quatre visites prénatales, avaient un risque de décès inférieur de 82% par rapport à ceux dont les mères n'avaient fait aucune visite [21]. La lutte contre les infections maternelles, la reconnaissance et le traitement des affections maternelles, auraient un impact positif sur les mortalités maternelle et néonatale. Le décès était aussi 2,9 fois plus fréquent chez les nouveau-nés dont les mères résidaient hors de la ville de Ouagadougou. Diallo et al. au Burkina Faso, avaient aussi retrouvé que les nouveau-nés résidant en campagne ou dans la périphérie de la ville avaient plus de risque de décéder que ceux vivant en ville [22]. L'inaccessibilité ou l'accès tardif aux centres de santé pouvant fournir des soins de qualité pourrait expliquer cette différence. Nous avons noté que le risque de décès était beaucoup plus élevé en 2011 (OR = 8,3) et 2012 (OR = 10) comparativement à 2009. Avec l'analyse de sensibilité, les OR associés à la variable année d'admission diminuent fortement si on émet l'hypothèse que les perdus de vue sont décédés. En effet, dans notre contexte, beaucoup de familles préfèrent ramener leurs malades pour qu'ils meurent à domicile lorsqu'ils estiment que les chances de survie sont nulles. Les fortes différences observées dans l'analyse principale seraient dues au fait qu'il y a eu plus de perdus de vue en 2009 (33 perdus de vue) qu'au cours des années suivantes (21 en 2010 et 2011 et 20 en 2012). Néanmoins, on note que les décès restent plus fréquents en 2011 et 2012 comparativement à 2009 même en considérant les perdus de vue comme décédés. Ces résultats sont corroborés par les données du service des archives et de documentation centrale du CHUP-CDG. Selon ces données, de 2009 à 2012, le nombre de décès néonatal était respectivement de 50, 71, 77 et 108 pour des admissions respectives de 565, 631, 551 et 702 [24]. Donc, entre 2009 et 2012, la mortalité aurait doublée alors que les admissions n'ont augmenté que du quart [25]. Cette augmentation de la fréquence des décès au cours des années reste à expliquer. Traduit-elle une détérioration de la qualité des soins? Si oui, cette diminution de la qualité des soins pourrait s'expliquer par une surcharge de travail du service de pédiatrie médicale. On a noté que le taux d'occupation des lits est passé de 51% en 2007 à 145% en 2010 [25].

Par ailleurs, comme dans bien des études [26, 27], le poids de naissance est un facteur de risque indépendant associé au décès néonatal puisqu'une augmentation de 100 g du poids de naissance étant associée à une diminution de 5% de la fréquence des décès. Dans une étude menée dans la ville de Ouagadougou au Burkina Faso, Ouédraogo-Yugbaré et al. avaient retrouvé aussi le faible poids de naissance comme un facteur associé au décès néonatal [28]. Une augmentation de 100 g du poids de naissance entraînerait une diminution significative de 5% du risque de décès néonatal.

## Conclusion

Une proportion importante de nouveau-nés est considérée comme infectée par une bactérie à l'admission au CHUP-CDG mais très peu bénéficieront d'un examen bactériologique de confirmation diagnostique. La prise en compte des facteurs associés au décès en milieu hospitalier et dans les structures sanitaires périphériques permettrait de réduire la mortalité néonatale. Aussi une étude de cohorte prospective avec des critères de suspicions d'infections bactériennes néonatales confirmés par la bactériologie permettraient d'identifier également des facteurs bactériologiques associés au décès des nouveau-nés.

### Etat des connaissances actuelle sur le sujet

- Le faible poids de naissance est associé à la mortalité néonatale.
- Les germes responsables de l'infection néonatale sont le streptocoque B, Escherichia. Coli et Listeria monocytogenès dans les pays développés.

### Contribution de notre étude à la connaissance

- La consultation prénatale, la réanimation à la naissance et le poids de naissance sont les facteurs associés au décès.
- Seuls 6,9% des prélèvements bactériologiques sont revenus positif.;
- *Escherichia. coli* et *Klebsiellapneumoniae* étaient isolés dans respectivement 34,4% et 17,2% cas.
